# Cytochrome P450 3A1 Mediates 2,2′,4,4′-Tetrabromodiphenyl Ether-Induced Reduction of Spermatogenesis in Adult Rats

**DOI:** 10.1371/journal.pone.0066301

**Published:** 2013-06-07

**Authors:** Zhan Zhang, Xiaoming Zhang, Zhenzhen Sun, Huibin Dong, Lianglin Qiu, Jun Gu, Jingping Zhou, Xinru Wang, Shou-Lin Wang

**Affiliations:** 1 State Key Lab of Reproductive Medicine, Institute of Toxicology, Nanjing Medical University, Nanjing, Jiangsu, P.R.China; 2 Key Lab of Modern Toxicology of Ministry of Education, School of Public Health, Nanjing Medical University, Nanjing, Jiangsu, P.R.China; Clermont Université, France

## Abstract

**Background:**

2,2′,4,4′-tetrabromodiphenyl ether (BDE47) is the dominant PBDE congener in humans, wildlife, and the environment. It has been reported to be metabolized by cytochrome P450 (CYP) enzymes. Still, the effects of BDE47 on spermatogenesis failure are attracting an increasing amount of attention. However, it is unclear whether CYP-mediated metabolism contributes to BDE47-induced reproductive toxicity.

**Methodology and Principal Findings:**

The role of cytochrome P450 3A1 (CYP3A1) in the formation of oxidative metabolites of BDE47 and its induced spermatogenesis failure was investigated in SD rats. BDE47 significantly increased the expression and activity of CYP3A1 in rat liver, and 3-OH-BDE47, the major oxidative metabolite of BDE47, dose-dependently increased in rat liver, serum, and testis, which was aggravated by dexamethasone (DEX), an inducer of CYP3A1. Additionally, testicular 3-OH-BDE47 and reactive oxygen species (ROS) in seminiferous tubules increased especially when BDE47 was administered in combination with DEX, which was confirmed in GC-1 and GC-2 cells that 3-OH-BDE47 induced more ROS production and cell apoptosis via the upregulation of FAS/FASL, p-p53 and caspase 3. As a result, daily sperm production dose-dependently decreased, consistent with histological observations in giant cells and vacuolar spaces and increase in TUNEL-positive apoptotic germ cells.

**Conclusion:**

CYP3A1-mediated metabolic activation of BDE47 and the active metabolite 3-OH-BDE47 and consequent ROS played an important role in reduction of spermatogenesis by germ cell apoptosis. Our study helps provide new insights into the mechanism of reproductive toxicity of environmental chemicals.

## Introduction

Polybrominated diphenyl ethers (PBDEs) are a class of flame-retardant chemicals frequently applied to textiles, furniture, and electronic and electrical items [1]. Currently, PBDEs are recognized as environmental pollutants of global concern because their levels in the environment and in humans have increased markedly over the past several decades. In China, the annual consumption of PBDEs has increased at an estimated rate of 8% since 2000, and the predominant product used is the deca-BDE technical mixture, amounting to 30,000 tons in 2005, followed by octa-BDE and penta-BDE [2], [3]. Evidence from several studies demonstrated that PBDEs are harmful to male reproduction. A previous study reported that developmental exposure to a single low dose (60 µg/kg.bw) of 2,2′,4,4′,5-pentabromodiphenyl ether (BDE99) decreased sperm count in male Wistar rats [4]. Further studies demonstrated that these environmental toxicants commonly increase oxidative stress by downregulating the production of antioxidant enzymes such as superoxide dismutase, catalase and glutathione peroxidase [5]. 2,2′,4,4′-tetrabromodiphenyl ether (BDE47), the dominant congener of PBDEs, is usually found in human blood and milk samples [6]. It is also the dominant congener to which humans, especially infants, are exposed via inhalation [7]. In animals, BDE47 has been identified as a developmental, reproductive, and neurological toxicant and disrupter of multiple endocrine systems [8]. Strong inverse correlations were found between the serum concentration of 2,2′,4,4′,5,5′-hexabromodipheny ether (BDE153) and sperm concentration and testis size [9]. Semen mobility was negatively related to BDE47 concentration in human blood plasma [10]. These results suggest that BDE47 might contribute to male reproductive dysfunction, such as spermatogenesis failure, but the mechanisms are still unclear.

Accumulating evidence from several studies has demonstrated that PBDEs are biotransformed to hydroxylated metabolites (OH-BDEs) in mammals. OH-BDEs have been detected in bile, urine, and feces in rats and mice treated with BDE47 [11] and in blood samples from women and children who were environmentally exposed to PBDEs [12]. The formation of OH-BDEs of BDE47 and BDE99 by rat and human hepatic cells and microsomes has been reported [13], [14], [15]. More recently, OH-BDEs have been regarded as a relatively new group of phenolic compounds that have attracted particular interest because of their biological effects, including disruption of thyroid hormone homeostasis and sex hormone steroidogenesis, which might mediate PBDE-induced reproductive toxicity [16], [17]. The cytochrome P450s (CYPs) enzyme is well known to represent the predominant biotransformation system because CYP catalyze the first step in oxidative metabolism, thereby serving as a significant determinant of the bioaccumulation of pollutants such as PBDEs. CYPs usually metabolize carcinogens to their inactive derivatives but occasionally convert the chemicals to more potent carcinogens [18]. BDE47 appeared to be able to upregulate cytochrome P450 1A (CYP1A), CYP2B, and CYP3A in F344 rats [19]. The incubation of BDE47 with rat hepatic microsomes or recombinant CYP enzymes generated a total of five OH-BDEs, among which 3-hydroxy-2,2′,4,4′-tetrabromodiphenyl ether (3-OH-BDE47) was the major metabolite, and CYP3A1 was assumed to exclusively mediate 3-OH-BDE47 formation [14]. However, very few studies have investigated the role of CYP and its mediation of the active metabolites of BDE47 in male reproductive dysfunction.

In this study, adult male rats were treated with a series of concentrations of BDE47 for 8 weeks. Then, the expression and activity of hepatic CYP3A1, and the metabolites of BDE47 in plasma, liver and testis were determined. Dexamethasone (DEX), an inducer of CYP3A1 expression, was used to be a tool to confirm the metabolic activation of CYP3A1 to BDE47. In addition, the testicular pathological observation, reactive oxygen species (ROS), apoptosis, spermatogenic processes, as well as the expression of apoptosis-related proteins were evaluated. The purpose of the study was to explore the role of CYP3A1 in BDE47-induced dysfunction of spermatogenesis, and the toxic process and related molecular mechanism. The results will help provide insights into the role of biotransformation in environmental toxicity and provide new clues to the prevention and control of environmental pollutant-induced biological damages.

## Materials and Methods

### Chemicals and reagents

BDE47 (purity of ≥ 98.7%) was purchased from Chemservice (West Chester, PA, USA). Dexamethasone phosphate (DEX), corn oil, acetic anhydride, and methyl tert-butyl ether were purchased from Sigma-Aldrich (St. Louis, MO, USA). Florisil SPE cartridge (500 mg, 6 ml, 100–200 mesh) was obtained from ANPEL Scientific Instruments (Shanghai, China). 3,3′,4,4′-tetrabromodiphenyl ether (BDE77), 3-OH-BDE47, 5-OH-BDE47, 6-OH-BDE47 and 4′-OH-BDE49 standard solutions were obtained from Accustandard (New Haven, CT, USA). Pyridine (anhydrous 99.5%) was purchased from Caledon Laboratories (Georgetown, Ontario, Canada). Hexane, toluene, dichloromethane (CH_2_Cl_2_), and isopropanol (chromatographically pure) were obtained from Merck (Darmstadt, Germany).

GC-1 and GC-2 cells were purchased from ATCC (Manassas, VA, USA). Dulbecco’s modified Eagle’s medium (DMEM), Williams’ Medium E, and collagen-coated culture plates were purchased from GIBCO-BRL (Grand Island, NY, USA). Antibodies specific for CYP3A1 were obtained from Millipore (Billerica, MA, USA). Antibodies specific for caspase-3, cleaved caspase-3, p53, *p*-p53 (ser15), PTEN, AKT, p-AKT (ser437), Bad, and an enhanced chemiluminescence (ECL) immunoblotting assay kit were purchased from Cell Signaling Technology (Danvers, MA, USA). Antibodies specific for FAS and FASL were purchased from Biosynthesis Biotechnology (Beijing, China). Antibodies specific for GAPDH, Cell Counting Kit-8 (CCK-8), Hoechst 33258, penicillin, streptomycin, trypsin-ethylenediaminetetraacetic acid (EDTA), the bicinchoninic acid (BCA) protein assay kit, catalase (CAT), and bovine serum albumin (BSA) were purchased from Beyotime Institute of Biotechnology (Shanghai, China).

### Determination of mRNA expression of CYP genes in rat primary hepatocytes treated with BDE47

Adult male Sprague Dawley rats (220–250 g) were purchased from Shanghai SLAC Laboratory Animal Co., Ltd (Shanghai, China). The primary hepatocytes were isolated following the two-step *in situ* collagenase perfusion method [20]. When the cells were prepared, they were treated with 10 µM BDE47 (dissolved in dimethyl sulfoxide [DMSO]) for 24 h for the determination of the induction of related *CYP* genes. To produce metabolites of BDE47, the hepatocytes were treated with 10 µM BDE47 once every 24 h for 3 days to take advantage of the potential increase in the formation of BDE47-related metabolites.

The expression of several genes that encode potential biotransforming enzymes, such as *CYP1A1*, *CYP1A2*, *CYP2B1/2*, *CYP2E1*, and *CYP3A1*, was determined using real-time quantitative reverse transcription polymerase chain reaction (qRT-PCR), and *GAPDH* was used as a reference to normalize expression levels. Primers were designed using Primer 5.0 software and synthesized by Invitrogen (Shanghai, China). The PCR primer sequences are shown in Table S1, and qRT-PCR was performed using FastStart Universal SYBR Green Master (Roche, Mannheim, Germany) on a 7300 Fast Real Time PCR System (Applied Biosystems, Life Technologies, Warrington, UK) following the manufacturer’s instructions. Relative gene expression was analyzed according to the 2^−ΔΔCt^ method [21].

### Animal treatment and sample collection

Male Sprague-Dawley rats (180–200 g) were obtained from Shanghai SLAC Laboratory Animal Co., Ltd (Shanghai, China). The animals were maintained on a 12 h/12 h light/dark cycle at ambient temperature (22°C) and 55% relative humidity. Two rats were placed in a plastic Macrolon cage with stainless steel covers and wood shavings, and sterilized food and tap water were provided ad libitum. The rats were allowed to acclimate for 1 week before the study began. Eighty rats were randomly divided into two groups: intraperitoneal injections of 5 ml/kg saline (Saline group; twice per week) and 10 mg (5 ml)/kg dexamethasone phosphate (DEX group; twice per week). Each group comprised four subgroups (10 rats per subgroup). The rats received a series of concentrations of BDE47 (0.001, 0.03, and 1 mg/kg/day) by intragastric administration for 8 weeks, 6 consecutive days per week. The control rats in each group were given the same volume of vehicle (0.5 ml corn oil). Twenty-four hours after the final treatment, the rats were euthanized by CO_2_ asphyxiation, followed by exsanguination via cardiac puncture for blood collection. The blood samples were centrifuged to obtain serum and plasma. The liver and testis were dissected and weighed (wet weight), and the organic coefficients were calculated. All of the samples were frozen and held at −80°C until analysis. This study was carried out in strict accordance with the recommendations in the guidelines of the Animal Care and Welfare Committee of Nanjing Medical University. The protocol was approved by the Committee on the Ethics of Animal Experiments of Nanjing Medical University, China. All surgery was performed under carbon dioxide anesthesia, and all efforts were made to minimize suffering.

### Determination of the expression and activity of CYP3A1 in rat liver

The frozen liver samples were homogenized (1:4, w/v) in ice-cold 0.1 M phosphate buffer (pH 7.4) that contained 1 mM EDTA. The homogenate was first centrifuged at 10,000 × *g* for 15 min at 4°C. The supernatant was then separated by cryogenic ultracentrifugation (Beckman Coulter, Indianapolis, IN, USA) at 100,000 × *g* for 60 min. The resulting microsomal pellets were finally suspended in a 0.25 M sucrose solution that contained 1 mM EDTA and stored at –80°C until use. After protein concentrations were detected using the BCA protein assay kit, CYP3A1 expression was determined using a specific antibody and an immunoblotting assay as described previously [22]. Hepatic CYP3A-related 7-ethoxyresorufin *O*-deethylase (EROD) activity was measured using a fluorescence assay kit (Genmed Scientifics, Shanghai, China) according to the manufacturer’s instructions. Briefly, 7-benzyloxyresorufin was used as the probe substrate for 7-benzyloxyresorufin *O*-dealkylatase (CYP3A1). The microsomal protein concentrations of the metabolic reactions were 80 µg/ml. Finally, the fluorescence intensity (λ_ex_  =  530 nm, λ_em_  =  590 nm) was read in an Infinite M200 plate reader (Tecan, Seestrasse, Männedorf, Switzerland).

### Determination of hydroxylated metabolites of BDE47 in primary hepatocytes and biological samples in rats

Sample extraction and cleanup were similar to a previous study [23]. Briefly, 1 ml of the cell culture medium from primary hepatocytes, 1 ml plasma, and 1 g of the liver or 1 g of the testis were put into a test tube. Internal standards (BDE77, 50 ng/sample) were added, vortexed, and left to equilibrate for 1 h. Hydrochloric acid and 2-propanol were then added to denaturize the proteins to release the lipids and the organohalogen compounds for the analyte extraction by n-hexane and methyl tert-butyl ether (1∶1). All of the samples were washed using florisil SPE cartridge eluted with 1∶1 CH_2_Cl_2_: hexane and then nitrogen evaporated to near dryness. After dissolving with 500 µl toluene, 100 µl pyridine and acetic anhydride were added to the sample, followed by vortexing for 2 min and heating at 60°C for 30 min. After derivatization, 700 µl water was added to pull out reaction. The sample was vortexed and back-extracted with hexane, nitrogen-evaporated to near dryness, and reconstituted in 100 µl CH_2_Cl_2_. The analysis of both BDE77 and hydroxylated metabolites of BDE47 was performed on a gas chromatograph-mass spectrometer (Thermo Finnigan DSQ, USA).

For the gas chromatography-mass spectrometry (GC-MS) analysis, a Voyager single quadrupole mass spectrometer equipped with a trace gas chromatograph and autosampler was used for the analysis of BDE47 metabolites as described previously [23]. A DB-5u (30 m×0.25 mm inner diameter × 0.25 µm film thickness; J&W Scientific, Folsom, CA) capillary column was used, with helium as the carrier gas at 1 ml/min. The temperature program was from 110°C for 1 min to 220°C for 1 min at 18°C/min, from 220°C to 240°C for 2 min at 8°C/min, and then to 300°C for 10 min at 8°C/min. One microliter of sample was injected using the splitless injection mode with a splitless time of 1 min. The injector, interface, and source temperatures were set at 260°C, 250°C, and 250°C, respectively. The mass spectrometer was operated under positive electron ionization mode with the filament in the trap stabilization mode at 70 eV. Acquisition was performed in full-scan mode over a mass range from 85 to 550 Da for the qualitative analysis. The data were acquired in the select ion monitoring mode (SIM) monitoring the molecular peak [M]^+^ for BDE77 and the [M-CH_2_O_2_Br]^+^ for OH-BDE47. Metabolite was identified by comparisons of its retention time and mass-to-charge ratio (m/z) values with authentic standards. Recovery was approximately 85–110%, and the relative standard deviation (RSD) was approximately 8%.

### Apoptosis assay in germ cell lines

The cells were cultured in DMEM supplemented with 10% fetal bovine serum (FBS), 2 mM L-glutamine, 100 U/mL penicillin, and 100 µg/mL streptomycin at 37°C in a humidified atmosphere that contained 5% CO_2_. For the apoptosis assay, GC-1 and GC-2 cells were treated with different concentrations of BDE47 or its metabolite 3-OH-BDE47 in six-well plates (5×10^5^ cells/well) for 24h. Cellular apoptosis was determined using Hoechst 33258 assay and flow cytometry assay (BD FACS Aria III, USA) as describe previously [22].

### Determination of reactive oxygen species in rat testis and germ cells

Testicular ROS were measured fluorimetrically with the probe 6-chloromethyl-2′,7′-dichlorodihyrofluorescin diacetate acetyl ester (CM-H2DCFDA; Genmed Scientifics, Shanghai,China) as described previously [24]. Briefly, rat testes were embedded in OCT-embedding compound (Tissue Tek; Sakura Finetek, Torrance, CA, USA), and 8 µm cross-cryosections were prepared. The cryosections were washed with PBS, incubated with CM-H2DCFDA at 37°C for 20 min, and washed again to remove excess CM-H2DCFDA. Subsequently, the cryosections were incubated with DAPI for 10 s, rinsed in PBS, mounted with glycerol: PBS (1: 9), and finally examined under a confocal Zeiss LSM710 microscope (Carl Zeiss, Hamburg, Germany). The fluorescence intensity was quantified using LSM Software. For ROS assay *in vitro*, GC-1 cells treated with BDE47, 3-OH-BDE47 or CAT were incubated with CM-H_2_DCFDA at 37°C for 20 min. Intracellular ROS production was detected using the fluorescence intensity of CM-H_2_DCFDA in a fluorescence microscope.

### Morphological observation of rat testis

After sacrifice, the testes of the rats were fixed in 4% neutral buffered formalin and dehydrated with graded ethanol. Each tissue was embedded in paraffin, and 5 µm cross-sections were prepared and stained with hematoxylin and eosin (H&E). The histopathological findings were investigated using an optical microscope (Axioskop 2 plus, Carl Zeiss, Hamburg, Germany). Photomicrographs of 10 fields per testis were taken at 200× magnification. Giant cells were identified by their characteristic features with multiple (≥2) round spermatid nuclei in a syncytial body present in the lumen. For each section of the testis, the total number of giant cells and seminiferous tubules was counted, and the average number of giant cells per tubule was calculated.

### Determination of daily sperm production in rats

Daily sperm production (DSP) was also determined according to previous studies [25]. Briefly, once the testis of the rats was decapsulated and weighed, it was homogenized in 5 ml of 0.9% NaCl that contained 0.5% Triton X-100 and 0.01% thiomersal sodium. After 10-fold dilution, a sample was transferred to a hemocytometer, and the number of mature spermatids was counted. DSP (sperm × 10^6^/g testis per day) was calculated by dividing the total number of mature spermatids per testis by 6.1 (i.e., the days of the seminiferous cycle that the spermatids are present in the seminiferous epithelium).

### Serum hormones analysis

[^125^I]-estradiol (E_2_), [^125^I]-follicle-stimulating hormone (FSH), [^125^I]-LH, and [^125^I] -testosterone (T) specific radioimmunoassay kits (Beijing North Institute of Biological Technology, Beijing, China) were used to determine the serum concentrations of E_2_, FSH, LH, and T according to the manufacturer’s instructions.

### Determination of apoptosis and related proteins in rat testis and germ cells

Immunochemistry was used to determine apoptosis (i.e., TUNEL-positive cells) in rat testis. Briefly, the testes were fixed in 4% neutral buffered formalin and paraffin-embedded, and 5 µm cross-sections were prepared. TUNEL labeling was performed using an In Situ Apoptosis detection kit, POD (Roche, Mannheim, Germany). Apoptotic indices were determined by counting the total number of TUNEL-positive cells. TUNEL-labeled sections were examined under 400× magnification, and the total number of tubules and apoptotic cells in 500 tubules was counted from at least three sections per testis per rat. Additionally, equal amounts of protein (100 µg for testis tissue; 75 µg for GC-1 cells) were separated by sodium dodecyl sulfate-polyacrylamide gel electrophoresis and transferred into a polyvinylidene fluoride membrane (Millipore, Bedford, MA). Using specific antibodies for FAS, FASL, p53, PTEN, AKT, Bad, and cleaved-caspase 3, the protein immune complexes were detected using an ECL immunoblotting assay kit and exposed to Kodak X-Omat film as described previously [22].

### Statistical analysis

Statistical differences were analyzed using one-way analysis of variance (ANOVA) and Student–Newman–Keuls multiple comparison test by SPSS 13.0 software (Chicago, IL, USA). The results are expressed as mean ± standard deviation (SD) or standard error (SE). Differences were considered statistically significant at *P≤*0.05.

## Results

### Induction of hepatic cytochrome P450 3A1 by treatment with BDE47

**Figure 1:**
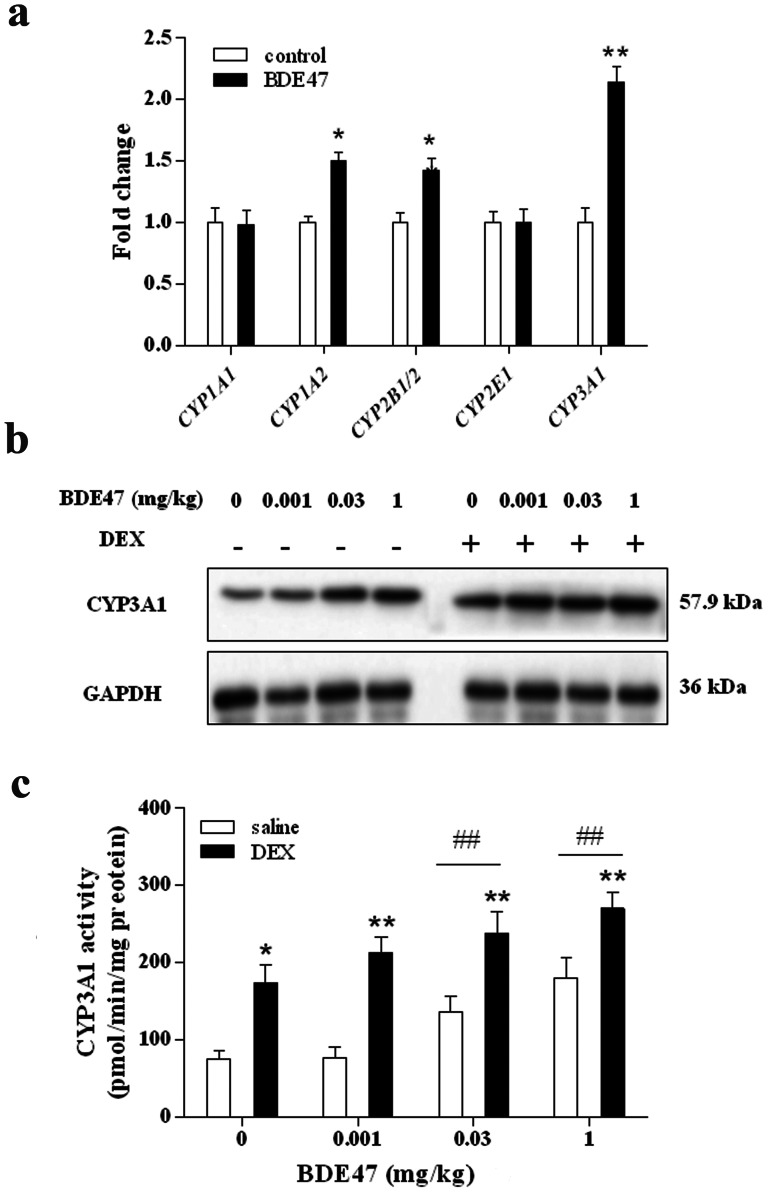
Induction of CYP3A1 by BDE47 in primary hepatocytes and liver in rats. **(a)** mRNA expression of several *CYP* genes that encode potential metabolic enzymes in primary rat hepatocytes treated with 10 µM BDE47. cDNA (0.1 µg) for each gene was used in the quantitative real-time RT-PCR. The data are expressed as the mean ± SD of three independent experiments with triplicate samples. **(b)** Expression of CYP3A1 protein in rat liver with BDE47 treatment in saline or dexamethasone (DEX). Hepatic microsome protein (10 µg) for each sample was used in the immunoblotting assay. GAPDH was used as an internal reference. **(c)** Hepatic CYP3A-related 7-ethoxyresorufin *O*-deethylase (EROD) activity induced by treatment with BDE47 in saline or DEX, assessed using a fluorescence assay. The data are expressed as the mean ± SD from five rats in each subgroup. ^##^ *P*<0.01, compared with corresponding vehicle control, *P<0.05, **P<0.01, compared with corresponding BDE47 treatment in saline group.

The major hepatic cytochrome P450s (CYPs), such as CYP1A1, CYP1A2, CYP2B1/2, CYP2E1, and CYP3A1, were selected to screen CYPs related to the induction by BDE47. As shown in Fig. 1a, 10 µM BDE47 significantly increased the expression of *CYP1A2*, *CYP2B1/2*, and *CYP3A1* in primary hepatocytes in rats (*P*<0.05 or *P*<0.01). CYP3A1 expression in BDE47-treated cells was approximately 2.24-fold higher than in the corresponding control, suggesting that CYP3A1 might be an important CYP enzyme related to BDE47 metabolism. To confirm these results, the expression and activity of hepatic CYP3A1 protein was determined in BDE47-treated rats. As expected, the expression of CYP3A1 was dose-dependently increased by BDE47 treatment, which was aggravated by DEX, an inducer of CYP3A1 expression (Fig. 1b). Similarly, BDE47 dose-dependently increased the activity of hepatic CYP3A1 in rats, and DEX enhanced this induction, with a significant difference between BDE47 and BDE47 + DEX for each treatment (*P*<0.05 or *P*<0.01). With high-dose treatment (0.03 and 1 mg/kg), CYP3A1 activity after both BDE47 and BDE47 + DEX treatment was higher than their corresponding controls (*P*<0.01; Fig. 1c). These results indicate that BDE47 induced the expression and activity of CYP3A1, which might be involved in BDE47 metabolism.

### Identification and distribution of BDE47 metabolites in rats

A Representative GC/MS chromatograms 3-OH-BDE47 from BDE47-treated primary hepatocytes, and mass spectra of BDE77 (internal standard) and 3-OH-BDE47, are shown in Fig S1. 3-OH-BDE47 was found in BDE47-treated primary rat hepatocytes (Fig. S1a). In rat plasma, 3-OH-BDE47 exhibited a dose-dependent increase with BDE47 treatment, and DEX strengthened the effects compared with BDE47 treatment alone (Table 1). Although 3-OH-BDE47 was only found in highest-dose treatment of BDE47 (1 mg/kg) in rat liver and testis, it increased significantly in BDE47+DEX treatment. For example, 3-OH-BDE47 was about 2.21 ng/g in rat testis in 1 mg/kg treatment of BDE47 whereas DEX generated more 3-OH-BDE47 (1.05 ng/g in 0.03 mg/kg treatment and 6.31 ng/g in 1 mg/kg treatment) compared with BDE47 treatment alone (Table 1). Although other metabolites (5-OH-BDE47, 6-OH-BDE47 or 4′-OH-BDE49) were detected in plasma or liver, they were not found in rat testis. In addition, DEX did not increase their levels in plasma or liver when compared with the treatment of BDE47 alone (Table S2, Table S3). Considering the above induction of CYP3A1 treated by BDE47 and its oxidative metabolite in rat testis, the results indicate that 3-OH-BDE47 might be an important metabolite of BDE47 that is associated with BDE47-induced male reproductive toxicity.

**Table 1:** Concentrations of 3-OH-BDE47 in rat plasma, liver and testis.

		plasma		liver		testis	
BDE47 (mg/kg)	*n*	saline	DEX	saline	DEX	saline	DEX
0	10	< LOD	< LOD	< LOD	< LOD	< LOD	< LOD
0.001	10	0.53±0.10	1.02±0.72	< LOD	0.68±0.43	< LOD	< LOD
0.03	10	0.72±0.51	1.46±0.28	< LOD	2.00±0.84	< LOD	1.05±0.94
1	10	36.89±13.92	73.31±22.20	3.27±0.83	10.32±4.55	2.21±1.78	6.31±2.23

The data are expressed as mean ± SD; *n*, animal number; LOD, limit of detection; DEX, dexamethasone.

### Comparison of BDE47-induced cell apoptosis and 3-OH-BED47 in germ cells

**Figure 2:**
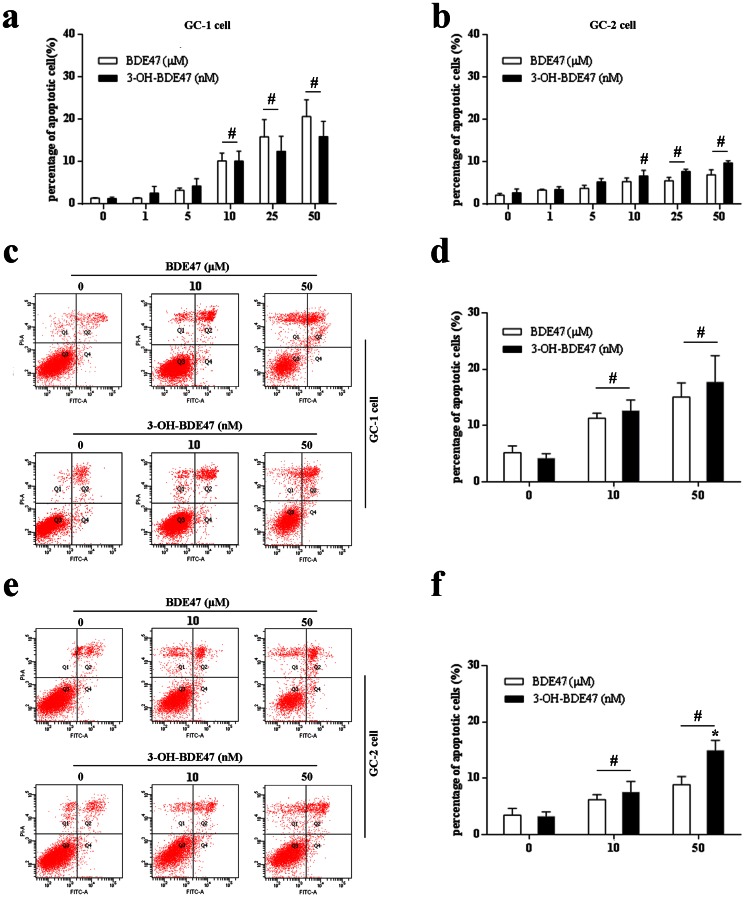
Cell apoptosis of BDE47 and 3-OH-BDE47 in GC-1 and GC-2 cells. Cell apoptosis were determined using the Hoechst 33258 assay and flow cytometry assay. **(a**–**b)** GC-1 cells or GC-2 cells were treated with BDE47 (0–50 µM) or 3-OH-BDE47 (0–50 nM) for 24h and then subjected to Hochest 33258 assay to detect apoptotic cell using fluorescence microscope. **(c**–**d)** Cell apoptosis of GC-1 cells treated with BDE47 (0, 10, 50 µM) or 3-OH-BDE47 (0, 10, 50 nM) for 24h and then subjected to flow cytometry assay. **(e**–**f)** Cell apoptosis of GC-2 cells treated with BDE47 (0, 10, 50 µM) or 3-OH-BDE47 (0, 10, 50 nM) for 24 h and then subjected to flow cytometry assay. The data are expressed as the mean ± SD of three independent experiments from triplicate samples. *P<0.05, compared with corresponding BDE47 treatment in saline group; ^#^ *P*<0.05, compared with corresponding vehicle control.

In the present study, 3-OH-BDE47 was the oxidative metabolite of BDE47 *in vivo*. To compare apoptosis induced by BDE47 or 3-OH-BDE47, both GC-1 and GC-2 cells were treated with different concentrations of BDE47 (0–50 µM) and 3-OH-BDE47 (0–50 nM) for 24 h. The results from Hoechst 33258 assay showed that both BDE47 and 3-OH-BDE47 dose-dependently induced apoptosis in GC-1 and GC-2 cells. GC-1 cells were more sensitive to treatment with BDE47 and 3-OH-BDE47 than GC-2 cells (Fig. 2a and 2b), which was confirmed by flow cytometry assay (Fig 2c–2f). Expectedly, 3-OH-BDE47 was much more toxic than BDE47 (about 1000-fold).

### Effects of BDE47 on spermatogenesis and serum testosterone levels

**Figure 3:**
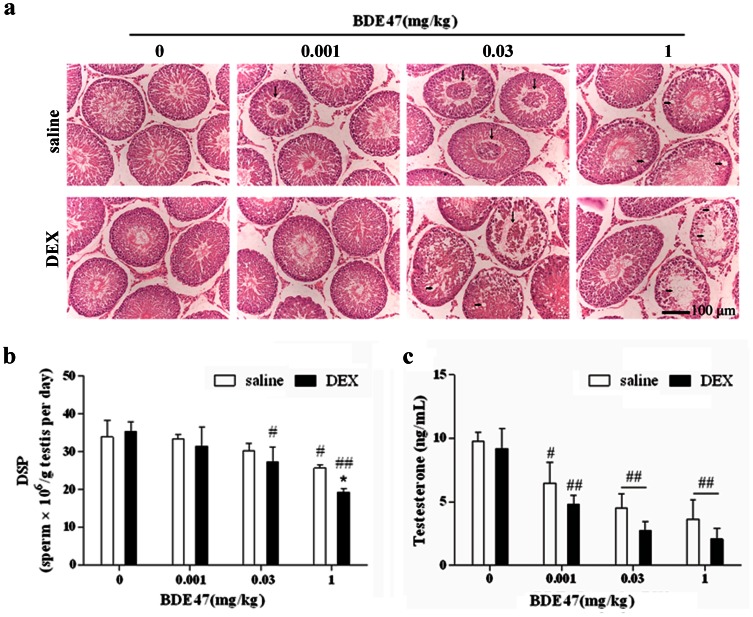
Effects of BDE47 on testicular morphological, daily sperm production and serum testesterone levels. **(a)** The testis tissue was stained with hematoxylin and eosin (H&E), and testicular morphological changes and disrupted seminiferous tubule architecture were assessed by optical microscopy. Horizontal arrow, giant cell; vertical arrow, vacuolar spaces in the epithelium. **(b)** Daily sperm production (DSP) in rats exposed to BDE47. **(c)** Serum testosterone (T) level in rats exposed to BDE47. Bars show the mean ± SD of 10 animals per group. *P<0.05, compared with corresponding BDE47 treatment in saline group; #*P* <0.05; ##*P*<0.01; compared with corresponding vehicle control.

The histological examination of the testes with rat exposure to BDE47 revealed a striking disruption of the normal cellular organization of the seminiferous epithelium. In the lumen, 0.03 and 1 mg/kg BDE47 significantly increased the number of multinucleated giant cells that arose from spermatocytes that aborted meiosis and appeared as a syncitium. Abundant vacuolar spaces in the seminiferous epithelium were found with high-dose BDE47 treatment, dexamethasone aggravated the effects of BDE47 on the morphological changes in the testis (Fig.3a). Following the above morphological changes, BDE47 decreased the number of spermatids and DSP. As shown in Fig. 3b, BDE47 dose-dependently decreased DSP, with a significant difference between the 1 mg/kg BDE47 treatment and control (*P*<0.05). As expected, DEX aggravated these detrimental effects, in which DSP was significantly lower in the DEX group than in the saline group (Fig. 3b). In addition, BDE47 significantly decrease serum testosterone level, and DEX aggravated this effect (Fig. 3c). However, other hormones, such as E_2_, FSH and LH, did not change after the treatments of BDE47 alone or combination with DEX (Fig.S2).

### Effects of BDE47 on the formation of ROS in seminiferous tubules

**Figure 4:**
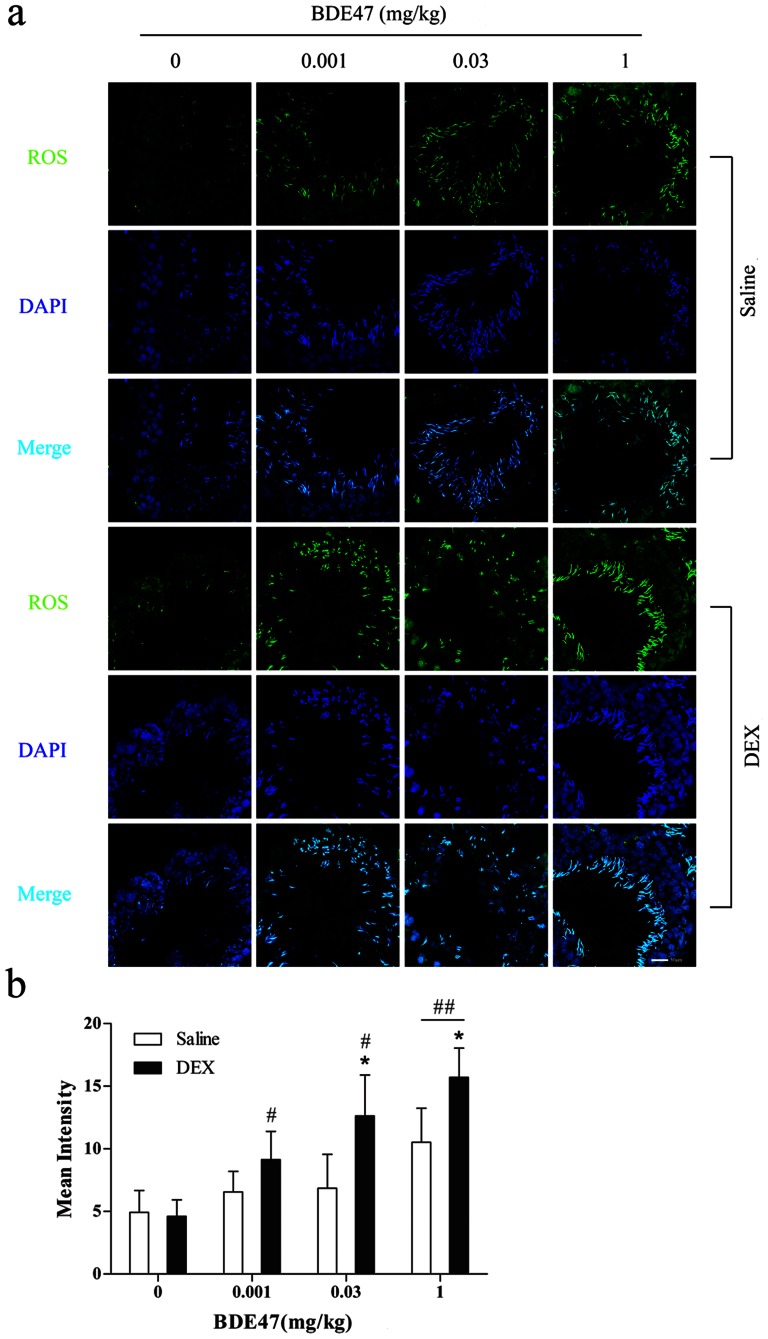
Production of reactive oxygen species (ROS) in seminiferous tubules in rats exposed to BDE47. Testicular ROS were measured fluorimetrically with the probe CM-H2DCFD (6-chloromethyl-2′,7′-dichlorodihyrofluorescin diacetate, acetyl ester). **(a)** Representative photograph of ROS in seminiferous tubules. **(b)** Fluorescence intensity quantified by LSM software. The data are expressed as the mean ± SD of five rats in each subgroup. *P<0.05, compared with corresponding BDE47 treatment in saline group; ^#^ *P*<0.05, ^##^ *P*<0.01, compared with corresponding vehicle control.

Reactive oxygen species were localized by immunofluorescence. The fluorescence in spermatozoa after BDE47 treatment (Fig. 4a) and the intensity increased dose-dependently with BDE47 dosage, in which 1 mg/kg BDE47 significantly elevated testicular ROS levels (*P*<0.05; Fig. 4b). As an inducer of CYP3A1, DEX increased fluorescence in the adluminal compartment at all stages of the epithelial cycle after BDE47 treatment (Fig. 4a), and the intensity also dose-dependently increased, in which testicular ROS levels with 0.001, 0.03 and 1 mg/kg treatment were 2-, 2.7-, and 3.4-fold higher than in controls, respectively (Fig. 4b). Moreover, DEX accelerated BDE47-induced ROS production. As shown in Fig. 4b, the fluorescence intensity was significantly higher with each dose of BDE47 in the DEX groups than in the saline group. These results indicate that BDE47 induced ROS formation, and CYP3A1 might mediate BDE47-induced ROS production.

### Effects of BDE47 or 3-OH-BDE47on the intracellular production of ROS and the expression of apoptosis-related proteins in GC-1 cells

**Figure 5:**
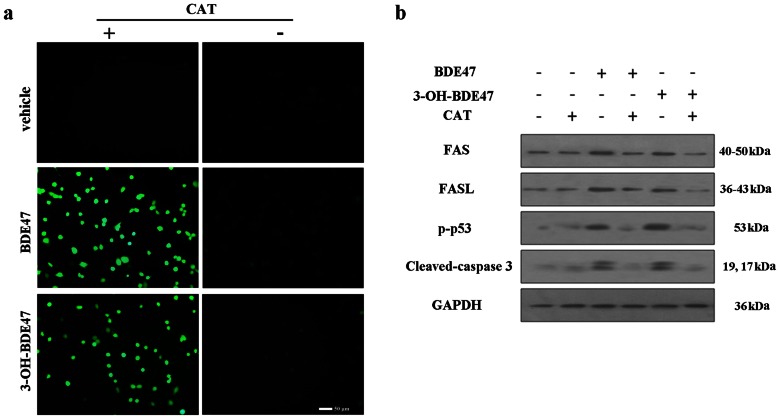
Effects of BDE47 on the production of intracellular ROS and the expression of apoptosis-related proteins in GC-1 cells. (a) Representative fluorescence images of ROS in GC-1 cells. GC-1 cells were incubated with BDE47 (10 µM) or 3-OH-BDE47 (10 nM) in presence or absence of 400 U/ml catalase (CAT) for 24h. (b) Expression of apoptosis-related proteins in GC-1 cells after BDE47 (10 µM), 3-OH-BDE47 (10 nM) in presence or absence of CAT (400 U/ml) for 24h using immunoblotting assay. GAPDH was used as an internal reference.

ROS have been reported to mediate apoptosis in a variety of cells. To investigate whether intracellular ROS production is involved in BDE47 or 3-OH-BDE47-induced cell apoptosis, GC-1 cells were treated with 10 µM BDE47 or 10 nM 3-OH-BDE47. As shown in Fig. 5a, BDE47, as well as 3-OH-BDE47, significantly increased intracellular ROS production, and upregulated the expression of p-p53, cleaved-caspase 3, FAS and FASL (Fig. 5b), all of which could be blocked by catalase (CAT), an antioxidant. The results suggested that ROS was an important mediator of apoptosis induced by BDE47 or 3-OH-BDE47.

### Effects of BDE47 on expression of apoptosis related proteins in rat testis

**Figure 6:**
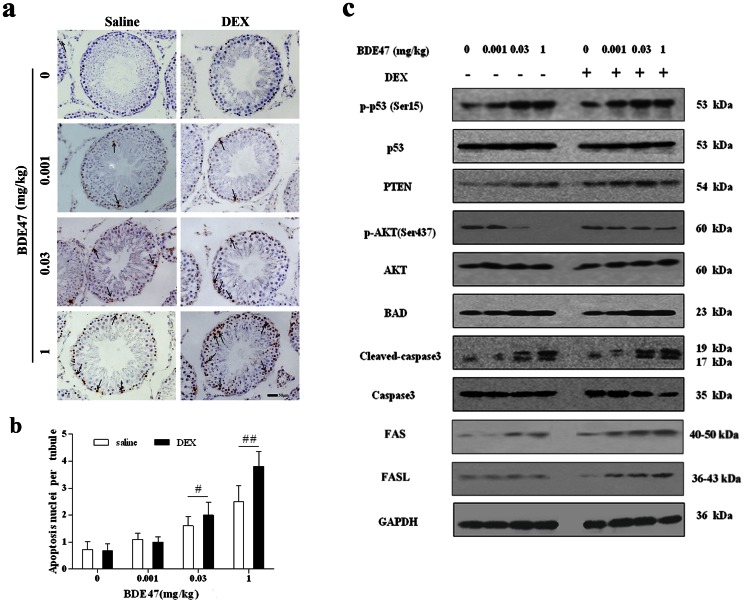
Effect of BDE47 on daily sperm production and germ cell apoptosis in seminiferous tubules. **(a)** TUNEL assay of germ cell apoptosis in rat testes. Arrows, apoptotic cells. **(b)** Average number of apoptotic nuclei per tubule. The data are expressed as the mean±SE of three rats per subgroup. **(c)** Expression of apoptosis-related proteins in rat testis after BDE47 treatment using immunoblotting assay. GAPDH was used as an internal reference. ^#^ *P*<0.05, ^##^ *P*<0.01, compared with vehicle control.

To estimate BDE47-induced apoptosis in the testis, TUNEL-positive cells in seminiferous tubules were counted. The number of TUNEL-positive germ cells increased in the BDE47 treatment groups, whereas controls had rare apoptotic cells (Fig. 6a). Apoptotic germ cells that formed after exposure to low-dose BDE47 were mostly restricted to the spermatogonia, but the apoptotic spermatocytes obviously appeared with 1 mg/kg BDE47 treatment in the DEX group (Fig. 6a). Quantitatively, BDE47 dose-dependently increased TUNEL-positive cells in the testis, with a significant difference for the 0.03 and 1 mg/kg BDE47 treatment compared with control (*P*<0.05; Fig. 6b). Dexamethasone aggravated these detrimental effects (Fig. 6b).

Based on the above results, apoptosis-related proteins in the testis were determined. As shown in Fig. 6c, p-p53 (Ser15) showed a dose-dependent increase, and DEX enhanced the increase in p-p53 expression. Previous studies showed that p53 protein regulated the transcriptional activation of the PTEN gene, confirming that BDE47 expectedly upregulated PTEN expression. PTEN has been reported to downregulate PI3K/Akt-dependent cell survival, and an obvious decrease in the expression of phospho-Akt (ser473) was observed after BDE47 treatment, suggesting that the inhibition of Akt phosphorylation might be related to germ cell survival. Additionally, BAD, a BCL-2 antagonist of cell death, is a BCL-2 homology domain 3 (BH3)-only proapoptotic BCL-2 family member that is inactivated by phosphorylation through Akt. As expected, BDE47 induced the overexpression of BAD and caused a subsequent increase in apoptosis with concomitant activation of caspase-3 (Fig. 6c). In addition, FAS and FASL were also upregulated by BDE47 (Fig. 6c). Dexamethasone magnified the changes in the proteins related to apoptosis induced by BDE47 (Fig. 6).

## Discussion

**Figure 7:**
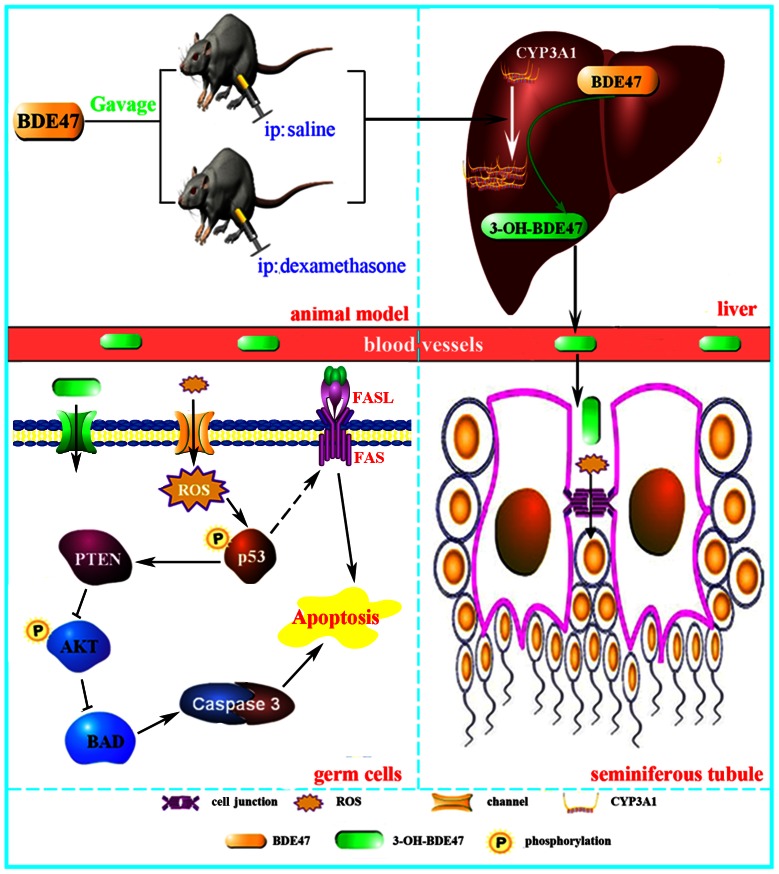
Schematic of the process and mechanism of CYP3A1-mediated BDE47-induced reduction of spermatogenesis.

The present study demonstrated that CYP3A1 plays an important role in the BDE47-induced reduction of spermatogenesis in adult rats. As shown in Fig. 7, BDE47 increased the expression and activity of CYP3A1, which subsequently metabolized BDE47 to its oxidative metabolites 3-OH-BDE47, and generated ROS. 3-OH-BDE47 showed much more toxicity than its parent compound, BDE47, and other types of metabolites, such as MeO-BDE47 [26]. Many environmental toxicants (e.g., cadmium chloride, methoxyacetic acid, and 1,3-dinitrobenzene) disrupt spermatogenesis by acting on the BTB [27]. And 3-OH-BDE47 might enter the microenvironment of the testis by disrupting integrity or function of blood-testis-barrier (BTB). 3-OH-BDE47 generated oxidative stress and apoptotic germ cells in seminiferous tubules via the FAS/FASL or p53/caspase 3 signaling pathway and contributed to the reduction of spermatogenesis. In addition, decrease serum testesterone might also contribute to the reduction of spermatogenesis [28]. Compared with BDE47, DEX, an inducer of CYP3A1, magnified the process and effects on spermatogenesis in rats. DEX induced more expression and activity of CYP3A1, formed more 3-OH-BDE47, generated more ROS and apoptosis, and aggravated the detrimental effects on spermatogenesis. Therefore, CYP3A1 mediated the oxidative metabolism of BDE47 and mediated the reduction of spermatogenesis.

Many studies have characterized the absorption, distribution, metabolite formation, and excretion of BDE47 [11], [29]. 3-OH-BDE47 had been identified in feces from rats that were exposed to BDE47 [11]. Similarly, 3-OH-BDE47 was detectable in plasma, liver, and testis in present study. 3-OH-BDE47 formation was reported previously to be mediated exclusively by CYP3A1 [14]. The CYP3A subfamily is the most important hepatic metabolic enzyme in the metabolism of many environmental toxicants. Kardem et al. reported that human CYP3A4 could mediate aflatoxin B1 (AFB1) to form its active metabolite AFB1-8,9-*exo*-epoxides to induce hepatic carcinogenesis [30]. In the present study, CYP3A1 was significantly induced not only in primary hepatocytes but also in rat liver, which was enhanced by DEX (positive CYP3A1 inducer), suggesting that CYP3A1 might be involved in BDE47 metabolism. Of the metabolites of BDE47, 3-OH-BDE47 was much more toxic than its parent BDE47. The ability of 3-OH-BDE47 to induce apoptosis was approximately 1000-fold higher than BDE47 in GC-1 or GC-2 cells, respectively, which significantly increased the toxicity of BDE47. These results indicate that hydroxylated PBDE metabolites (HO-PBDEs) might mediate their toxicity, but they are less persistent than their parents, and 3-OH-BDE47 mediated by CYP3A1 likely plays the critical role in the reproductive dysfunction observed in rats in this study.

BDE47 was reported to cause oxidative stress in different cells, such as RTG-2 cells [31], rainbow trout cells [32], Jurkat cells [33], and human fetal liver hematopoietic stem cells [34]. These effects are more pronounced with its hydrolated metabolites, such as 3-OH-BDE47. The present study showed that 10 nM 3-OH-BDE47 generated the similar production of intracellar ROS to10 µM BDE47 (1000-fold) in GC-1 cells, suggesting that 3-OH-BDE47 induced many more apoptotic cells in germ cells than BDE47 itself. CYP functions within a catalytic cycle, with iron undergoing changes in its spin state, oxidation number, and ligand coordination number as it proceeds around the ordered cycle. CYPs always mediate hydrolated metabolites and generate ROS. In the present study, the levels of ROS were significantly elevated in seminiferous tubules in rats exposed to BDE47, and more ROS were generated in rats in which CYP3A1 was induced by DEX. A recent study showed that OH-BDE47 increased intracellular ROS levels, reflected by GSH depletion and an elevation of SOD levels [26], suggesting that testicular ROS might be associated with 3-OH-BDE47 mediated by CYP3A1. OH-BDE47 significantly and dose-dependently increased micronucleus levels and apoptosis rate. Moreover, treatment with 6-OH-BDE47 resulted in marked cell cycle blockade [26]. Normal testicular spermatogenesis and steroidogenesis are generally sources of ROS. Although physiological levels of ROS are needed for spermatogenesis, excess ROS that results from environmental contaminants can have deleterious effects. In the present study, vacuolar spaces in the epithelium and giant cells were observed. The appearance of such giant cells is linked to defective spermatogenesis in genetically altered mice and human pathologies linked to infertility [35]. Similar to the ROS results, apoptotic germ cells appeared in rats treated with BDE47, especially in rats that were additionally exposed to DEX, both of which might result from the redundant formation of 3-OH-BDE47 mediated by CYP3A1.

In present study, BDE47 or 3-OH-BDE47 treatment significantly induced germ cells apoptosis, which was blocked by catalase, an antioxidant, suggesting that ROS was an apoptosis inducer and mediated the apoptosis via the activation of p53 that could control both FAS/FASL and caspase-dependent apoptosis [36], [37], [38]. In animals and GC-1 cells, p53 was activated by BDE47, and activation of p53 might be responsible for the upregulation of FAS and FASL. In addition, phosphatase and tensin homolog (PTEN) is induced by p53 and, in turn, stabilizes p53 by protecting it from mdm2-mediated ubiquitination [39]. In the present study, PTEN was upregulated after BDE47 treatment, which was enhanced by DEX showing increased toxicity. This positive feedback loop contributes to p53-induced apoptosis and senescence. PTEN, typically in the cytoplasm, is a major participant in the downregulation of AKT activity. An impairment of PTEN expression leads to an unhindered increase in AKT activity, which subsequently initiates downstream prosurvival effectors that lead to reduced apoptosis and increased cell proliferation [40]. When Bad is phosphorylated by AKT, it fails to perform its proapoptotic tasks. PTEN inhibited the phosphorylation of AKT, resulting in the dephosphorylation of BAD. Overexpression of BAD then activates caspase-3 to execute apoptosis.

In conclusion, we revealed a dominant contribution of CYP3A1 in BDE47-induced testicular toxicity. BDE47 increased the expression and activity of CYP3A1, which subsequently metabolizes BDE47 to form its oxidative metabolite, 3-OH-BDE47. 3-OH-BDE47 and consequent ROS disrupted seminiferous tubule architecture. This then induced germ cell apoptosis through the FAS/FASL or p53/caspase 3 signaling pathway, resulting in a reduction of spermatogenesis. This study provides new insights into the study of the mechanism of reproductive toxicity induced by environmental pollutants, especially environmental endocrine disruptors or POPs, providing important evidence for the prevention of reproductive hazards, control, and risk assessment, especially with low concentrations in the environment.

## Supporting Information

Figure S1 **Determination of 3-OH-BDE47 using gas chromatography-mass spectrometry mass spectra.** (a) Representative GC/MS chromatograms of BDE-77 (internal standard) and 3-OH-BDE47 from BDE47-treated primary hepatocytes in a DB-5 column under optimized conditions. The retention time of each standard is on the top of the chromatographic peaks. (b) Mass spectra of BDE77. The ion monitored for the BDE77 is [M]^+^. (c) Mass spectra of 3-OH-BDE47. During the ionization process of the OH-BDEs the acetyl group is lost, resulting in the [M-CH_2_CO]^+^ as the base peak. The concentration of the solution used in the analysis was spectra 500 ng/ml.
(TIF)

Figure S2 **Effect of BDE47 on serum reproductive hormones in rats.** (a) estrogen, (b) luteinizing hormone (LH), (c) follicle-stimulating hormone (FSH). Bars show the mean values ± SD of 10 animals per group.
(TIF)

Table S1 **Primers set for quantitative real-time RT-PCR analysis.**
(DOCX)

Table S2 **Concentrations of metabolites of BDE47 in rat plasma**
(DOCX)

Table S3 **Concentrations of metabolites of BDE47 in rat liver**
(DOCX)
