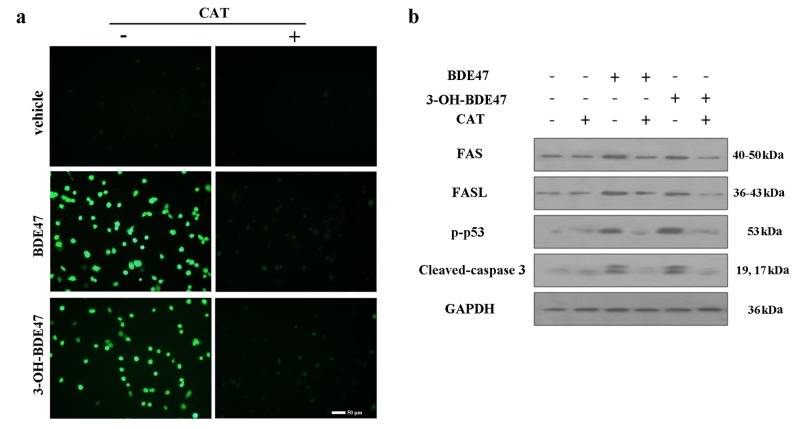# Correction: Cytochrome P450 3A1 Mediates 2,2′,4,4′-Tetrabromodiphenyl Ether-Induced Reduction of Spermatogenesis in Adult Rats

**DOI:** 10.1371/annotation/171e70e3-58e6-4d14-a361-1bc3a9692d46

**Published:** 2014-01-03

**Authors:** Zhan Zhang, Xiaoming Zhang, Zhenzhen Sun, Huibin Dong, Lianglin Qiu, Jun Gu, Jingping Zhou, Xinru Wang, Shou-Lin Wang

There is an error in the label of Figure 5a. The -/+ symbols underneath the CAT label have been switched. Please find a corrected version of the figure here: 

**Figure:**