# Supplementary figures and images for: Cytochrome P450 3A1 Mediates 2,2′,4,4′-Tetrabromodiphenyl Ether-Induced Reduction of Spermatogenesis in Adult Rats
Source: PLoS One. 2013 Jun 7;8(6):e66301. doi: 10.1371/journal.pone.0066301 (PMC3676375; doi:10.1371/journal.pone.0066301)

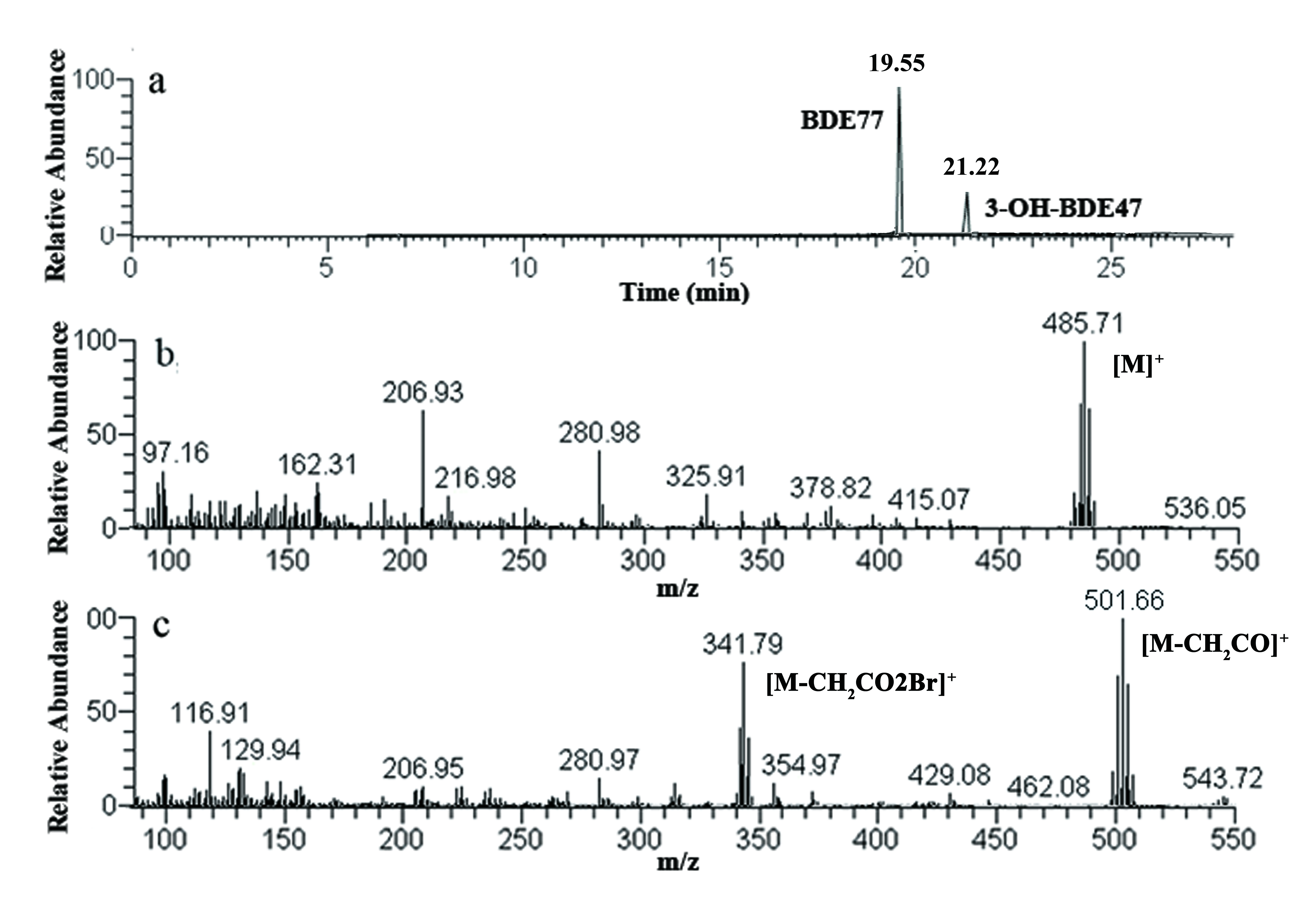

Supplement: Figure S1 — Determination of 3-OH-BDE47 using gas chromatography-mass spectrometry mass spectra. (a) Representative GC/MS chromatograms of BDE-77 (internal standard) and 3-OH-BDE47 from BDE47-treated primary hepatocytes in a DB-5 column under optimized conditions. The retention time of each standard is on the top of the chromatographic peaks. (b) Mass spectra of BDE77. The ion monitored for the BDE77 is [M]+. (c) Mass spectra of 3-OH-BDE47. During the ionization process of the OH-BDEs the acetyl group is lost, resulting in the [M-CH2CO]+ as the base peak. The concentration of the solution used in the analysis was spectra 500 ng/ml. (TIF) [file pone.0066301.s001.tif]

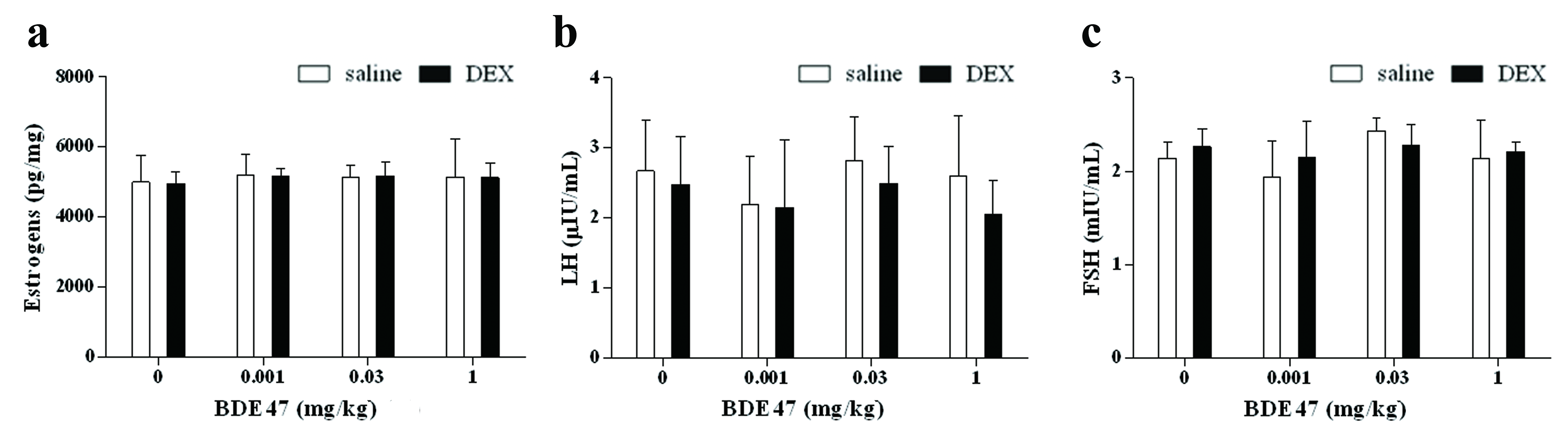

Supplement: Figure S2 — Effect of BDE47 on serum reproductive hormones in rats. (a) estrogen, (b) luteinizing hormone (LH), (c) follicle-stimulating hormone (FSH). Bars show the mean values ± SD of 10 animals per group. (TIF) [file pone.0066301.s002.tif]
