# Supplementary material for: Cytochrome P450 3A1 Mediates 2,2′,4,4′-Tetrabromodiphenyl Ether-Induced Reduction of Spermatogenesis in Adult Rats
Source: PLoS One. 2013 Jun 7;8(6):e66301. doi: 10.1371/journal.pone.0066301 (PMC3676375; doi:10.1371/journal.pone.0066301)
Supplement: Table S1 — Primers set for quantitative real-time RT-PCR analysis. (DOCX) [file pone.0066301.s003.docx]

**Table S1.** **Primers set for quantitative real-time RT-PCR analysis.**

| Gene | Accession no. | Primer sequence |
| --- | --- | --- |
| *CYP1A1* | X00469 | Forward: 5’-GGGTGTAGCACCTTCATTTAC-3’ |
|  |  | Reverse: 5’-GTTCAGAGGCAACTTGGACTA-3’ |
| *CYP1A2* | NM_012541 | Forward: 5’-TTTGGAGCTGGATTTGAAACAGT-3’ |
|  |  | Reverse: 5’-TCATGAATCTTCCTCTGCACCTT-3’ |
| *CYP2B1/2* | NM_37134 | Forward: 5’-CCCAATGTTTGGTGGAGGAA-3’ |
|  |  | Reverse: 5’-CTGTGATGCACTGGAAGAGGAA-3’ |
| *CYP2E1* | NM_031543 | Forward: 5’-GTGGTCCTGCATGGCTACA-3’ |
|  |  | Reverse: 5’-ACCTCCGCACATCCTTCC-3’ |
| *CYP3A1* | NM_173144 | Forward: 5’-CAGCAGCACACTTTCCTTTGTC-3’ |
|  |  | Reverse: 5’-CTCCTCCTGCAGTTTCTTCTGTGTA-3’ |
