# Supplementary material for: Cytochrome P450 3A1 Mediates 2,2′,4,4′-Tetrabromodiphenyl Ether-Induced Reduction of Spermatogenesis in Adult Rats
Source: PLoS One. 2013 Jun 7;8(6):e66301. doi: 10.1371/journal.pone.0066301 (PMC3676375; doi:10.1371/journal.pone.0066301)
Supplement: Table S2 — Concentrations of metabolites of BDE47 in rat plasma (DOCX) [file pone.0066301.s004.docx]

**Table S2. Concentrations of metabolites of BDE47 in rat plasma**

|  | |  | 6-OH-BDE47 | |  | 4’-OH-BDE49 | |
| --- | --- | --- | --- | --- | --- | --- | --- |
| BDE47 (mg/kg) | | n | saline | DEX |  | saline | DEX |
| 0 | 10 | | < LOD | < LOD |  | < LOD | < LOD |
| 0.001 | 10 | | 0.50±0.23 | 0.64±0.24 |  | 2.04±0.54 | 1.38±0.38 |
| 0.03 | 10 | | 0.40±0.14 | 0.37±0.07 |  | 2.53±0.24 | 2.42±0.38 |
| 1 | 10 | | 2.32±1.97 | 1.41±0.85 |  | 26.26±9.97 | 17.18±1.42 |

The data are expressed as mean ± SD, ng/g wet weight or ng/ml plasma; *n*, animal number; LOD, limit of detection; DEX, dexamethasone.
