# Supplementary material for: Cytochrome P450 3A1 Mediates 2,2′,4,4′-Tetrabromodiphenyl Ether-Induced Reduction of Spermatogenesis in Adult Rats
Source: PLoS One. 2013 Jun 7;8(6):e66301. doi: 10.1371/journal.pone.0066301 (PMC3676375; doi:10.1371/journal.pone.0066301)
Supplement: Table S3 — Concentrations of metabolites of BDE47 in rat liver (DOCX) [file pone.0066301.s005.docx]

**Table S3. Concentrations of metabolites of BDE47 in rat liver**

|  |  | 5-OH-BDE47 | |  | 4’-OH-BDE49 | |
| --- | --- | --- | --- | --- | --- | --- |
| BDE47 (mg/kg) | n | saline | DEX |  | saline | DEX |
| 0 | 10 | < LOD | < LOD |  | < LOD | < LOD |
| 0.001 | 10 | < LOD | < LOD |  | < LOD | < LOD |
| 0.03 | 10 | 1.07±0.18 | < LOD |  | < LOD | < LOD |
| 1 | 10 | 5.93±3.03 | 3.75±1.59 |  | 1.76±0.55 | 1.85±0.50 |

The data are expressed as mean ± SD, ng/g wet weight or ng/ml plasma; *n*, animal number; LOD, limit of detection; DEX, dexamethasone.
